# Evaluation of bone growth around bioactive glass S53P4 by scanning acoustic microscopy co-registered with optical interferometry and elemental analysis

**DOI:** 10.1038/s41598-023-33454-y

**Published:** 2023-04-24

**Authors:** Axi Holmström, Antti Meriläinen, Jere Hyvönen, Anton Nolvi, Tuomo Ylitalo, Kari Steffen, Robert Björkenheim, Gustav Strömberg, Heikki J. Nieminen, Ivan Kassamakov, Jukka Pajarinen, Leena Hupa, Ari Salmi, Edward Hæggström, Nina C. Lindfors

**Affiliations:** 1 Electronics Research Laboratory, Department of Physics, University of Helsinki, Helsinki, Finland; 2 Department of Orthopaedics and Traumatology, Department of Surgery, Helsinki University Hospital, University of Helsinki, Helsinki, Finland; 3 Department of Hand Surgery, Department of Surgery, Helsinki University Hospital, University of Helsinki, Helsinki, Finland; 4 Medical Ultrasonics Laboratory (MEDUSA), Department of Neuroscience and Biomedical Engineering, Aalto University, Espoo, Finland; 5 Department of Plastic Surgery, Department of Surgery, Helsinki University Hospital, University of Helsinki, Helsinki, Finland; 6 Johan Gadolin Process Chemistry Centre, Åbo Akademi University, Turku, Finland

**Keywords:** Ultrasound, Microscopy, Bone quality and biomechanics, Bone, Imaging techniques, Acoustics

## Abstract

Bioactive glass (BAG) is a bone substitute that can be used in orthopaedic surgery. Following implantation, the BAG is expected to be replaced by bone via bone growth and gradual degradation of the BAG. However, the hydroxyapatite mineral forming on BAG resembles bone mineral, not providing sufficient contrast to distinguish the two in X-ray images. In this study, we co-registered coded-excitation scanning acoustic microscopy (CESAM), scanning white light interferometry (SWLI), and scanning electron microscopy with elemental analysis (Energy Dispersive X-ray Spectroscopy) (SEM–EDX) to investigate the bone growth and BAG reactions on a micron scale in a rabbit bone ex vivo. The acoustic impedance map recorded by the CESAM provides high elasticity-associated contrast to study materials and their combinations, while simultaneously producing a topography map of the sample. The acoustic impedance map correlated with the elemental analysis from SEM–EDX. SWLI also produces a topography map, but with higher resolution than CESAM. The two topography maps (CESAM and SWLI) were in good agreement. Furthermore, using information from both maps simultaneously produced by the CESAM (acoustic impedance and topography) allowed determining regions-of-interest related to bone formation around the BAG with greater ease than from either map alone. CESAM is therefore a promising tool for evaluating the degradation of bone substitutes and the bone healing process ex vivo.

## Introduction

Bone substitutes are commonly used in orthopaedic surgery when bone tissue is missing due to trauma, infection, or bone tumour surgery. The most commonly used bone substitutes are autograft bone, allograft bone, and different kinds of synthetic bone substitutes, such as bioactive glass (BAG), calcium sulphate, ß-tricalcium phosphate (ß-TCP), hydroxyapatite (HA) or (ß-TCP)/(HA)-based bone substitutes^1^. The expected biological response of the implanted material is integration with bone followed by gradual remodelling or dissolution of the bone substitute with simultaneous bone ingrowth. Eventually, the implanted material should be replaced by bone.

BAG, invented by Larry Hench and co-workers in the late 1960s, is a synthetic silica-based bone substitute with proven bone bonding, osteoconductive, and osteostimulative properties^2^. The bone-formation-supporting characteristics of BAG are known to depend on controlled dissolution and precipitation processes at the surface of the BAG starting immediately after implantation. A rapid ion exchange of alkalis at the surface of the glass to hydrogen ions in the solution, followed by dissolution and repolymerization of soluble silica (Si), results in a Si-rich layer. Finally, calcium phosphate (CaP) nucleates and crystallizes to hydroxyapatite on top of the Si-layer at the surface of the BAG^3^.

BAG-S53P4 (in wt.%: 53% SiO_2_, 23% NaO, 20% CaO and 4% P_2_O_5_) has documented osteoconductive, osteostimulative, angiogenetic, and antibacterial properties^4–7^. Antimicrobial overuse has led to increased antibiotic resistance worldwide, and therefore, new innovations to combat infections are needed. The verified antibacterial properties of BAG-S53P4 makes it a promising tool in bone infection treatment. Due to its chemical reactions at the glass surface with subsequent elevation of pH and osmotic pressure, BAG-S53P4 has been shown to kill both planktonic bacteria and bacteria in biofilm^8^, thus making it one of the most interesting and promising bone substitutes to combat infections. Clinically, BAG-S53P4 is used in bone cavity filling and infection treatment in orthopaedic surgery^9–11^.

Radiographs are a common approach to characterize bone healing. However, as the HA-layer forming around BAGs resembles bone mineral, the contrast between the bone substitute and bone, and subsequently the evaluation of bone formation, is limited using current clinical applications, such as X-ray and computed tomography (CT). During the bone healing process, while new bone forms and the bone substitute disappears, the treated region appears more and more dense on X-rays and CT, making evaluation of the bone remodelling area difficult. Furthermore, studies have shown that bone mass density, determined from degree of mineralised bone as measured by synchrotron-radiation µCT, does not correlate significantly with elastic properties of bone^12,13^. Acoustic impedance, measured by scanning acoustic microscopy (SAM), is a more reliable measure^12,13^.

Scanning Acoustic Microscopy (SAM) employs highly-focused scanning ultrasound (US) at frequencies typically in the range of 10 MHz–2 GHz^12,14–16^. Since sound is a travelling density disturbance, the wave propagation of US is associated with the elasticity of materials with which it interacts. US, therefore, provides a mechanical contrast mechanism for imaging. More explicitly, in SAM, at each measured point, an ultrasonic pulse is transmitted from a focused transducer towards the sample and the reflected echo recorded. The Time-of-Flight of the echo provides the distance to the sample and allows constructing a topography map of the sample surface. The amplitude of the reflected echo, on the other hand, is determined by the difference in acoustic impedance of the imaging medium (typically water, *Z* = 1.5 MRayl) and the surface material (provided that the sample is flat and non-scattering). The acoustic impedance *Z* is the product of a material’s density, ρ, and speed of sound, *c*, (Z = ρ*c*) and is also related to the material stiffness *C* as *C* = Z*c*. The unit of *Z* is Rayl = kg/(m^2^ ∙s). The amplitudes of the reflected echoes in SAM thus provide an acoustic impedance map. Therefore, SAM has been used to spatially map elasticity-associated properties of materials, such as acoustic impedance, as well as shear and bulk moduli^15,17^. Practical bone-related applications include spatial characterization of bone^12,13,18–21^ and cartilage^22–24^.

The lateral resolution of SAM is limited by diffraction, typically enabling microscopic resolutions of 2–23 µm^12,19,20,25^. In SAM surface imaging (as opposed to subsurface imaging), axial resolution is dependent on the accuracy of determining the Time-of-Flight of the received echoes, provided that the surface is in focus. Usually, short pulses are used to obtain high axial resolution. Unfortunately, short pulses contain little energy, and hence the amplitudes of received echoes are low. This decreases the signal-to-noise ratio (SNR) of the acoustic impedance map, which is problematic for imaging soft samples. Utilizing coded excitation in SAM, i.e., transmitting long signals (to deposit more energy) with modulated frequency content (to allow accurately determining the Time-of-Flight), improves both the axial resolution and signal-to-noise ratio (SNR)^26^. With coded-excitation scanning acoustic microscopy (CESAM), features as small as ~ 150 nm in height can be distinguished on a surface^26^. CESAM could therefore provide an improved imaging modality, especially for bone-related samples, which contain both hard materials and soft tissue.

Scanning White Light Interferometry (SWLI) is a rapid contactless and label-free optical microscopy imaging method that can resolve topographical heights of spatially large areas down to sub-nanometer scale^27^*.* It relies on the short coherence length of white light to compare the optical distances from a light source to the sample and to a reference mirror^28^. The sample is imaged by scanning axially with a piezo actuator and recording images as a function of height. The recorded light-intensity changes from each pixel are then calculated with surface detection algorithms and a 3D topographical map of the sample is generated^29^*.* SWLI is feasible for imaging bio-related samples, such as oral implants^30^ and printed drug-laden polymer structures^31^. The spatial resolution of the SWLI is in practice mainly limited by the objective magnification. Higher magnifications can be used to gain higher resolution down to the diffraction limit, but the imaging area of a single scan is then reduced. Stitching methods can be used to combine partially overlapping multiple sub-aperture scans into a single large image^32^. This way, high spatial resolution can be achieved, while maintaining large area coverage. As with SAM, the maximum achievable lateral resolution is limited by the diffraction limit to approximately half of the centre wavelength of the used light^33^. As the wavelength of light (400–700 nm) is much shorter than that of ultrasound (6 µm in this study), SWLI is less sensitive to surface roughness and local inclinations in the sample surface.

The aim of this study was to determine the feasibility of our custom-built coded-excitation SAM (CESAM)^26,34,35^ for discerning differences in mechanical properties in the BAG structure and surrounding bone tissue in a leporine bone sample. This was achieved by co-registering the measured acoustic impedance with elemental analysis of SEM–EDX and the CESAM topography map with SWLI. All three techniques require an ex vivo bone sample, but studying bone formation ex vivo can still provide valuable insight into remodelling mechanisms and stages. The results suggest that CESAM could allow investigating bone healing with BAG ex vivo. The acoustic impedance map of CESAM is, however, sensitive to surface roughness, as the amplitude and frequency content of the reflected echoes can be distorted due to scattering. SWLI, which operates at shorter wavelengths than SAM, was used to validate the CESAM surface topography map. The topography maps were compared to evaluate the possibility of using only the CESAM topography map to determine scattering regions, i.e., regions with higher uncertainty in acoustic impedance. Furthermore, utilizing information from both the acoustic impedance and topography maps generated by the CESAM allowed determining regions-of-interest related to bone formation around the BAG with greater confidence. CESAM, therefore, can serve as a stand-alone tool for investigating bone formation ex vivo.

## Results

**Figure 1:**
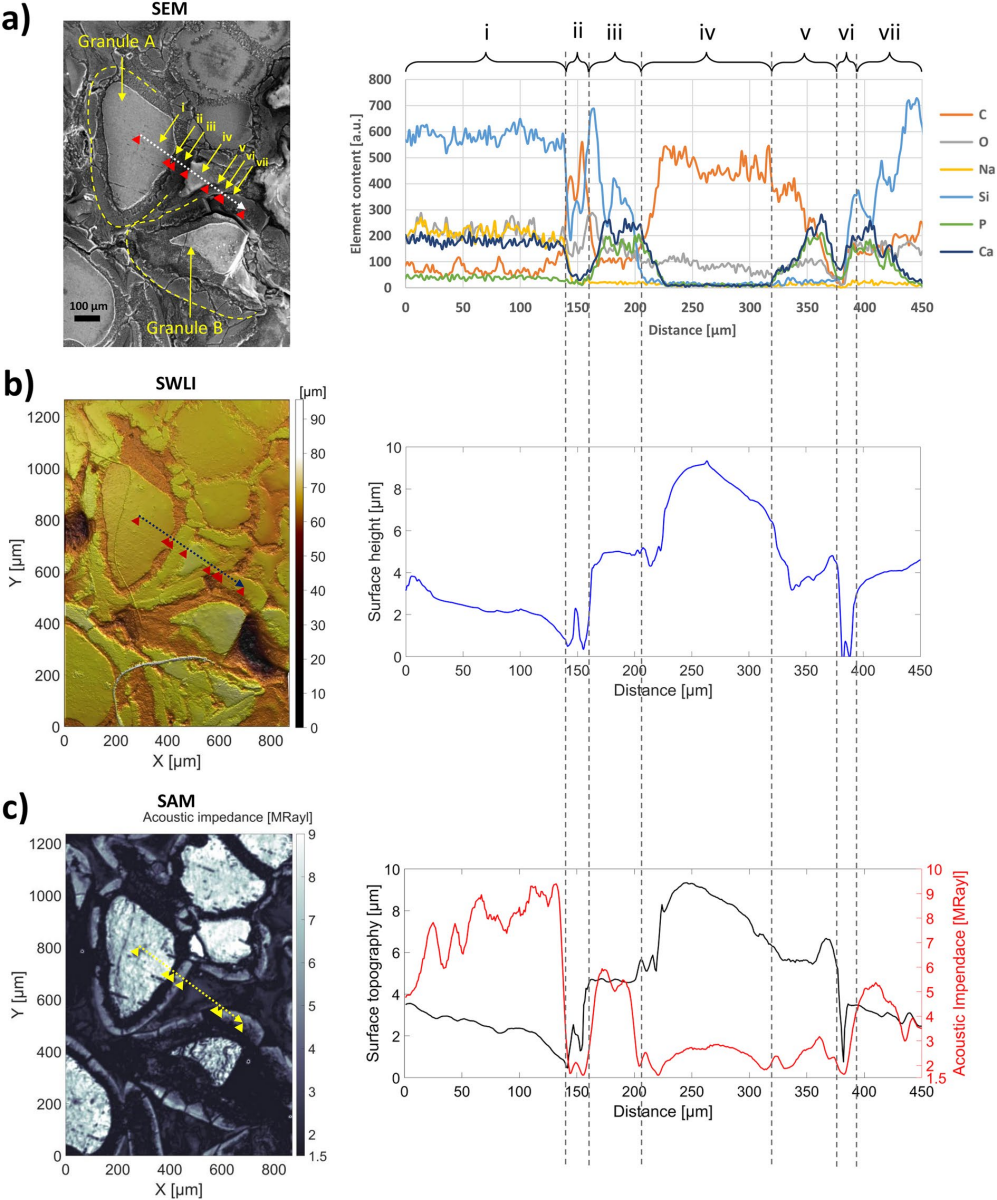
Bone growth measured with SEM–EDX (**a**), SWLI (**b**), and CESAM (**c**). (**a**) SEM image of BAG granules A and B (left) and EDX elemental analysis of the content along the indicated scan line (right). (**b**) SWLI image of the sample (left) and topography along the scan line (right). (**c**) CESAM acoustic impedance map of the sample (left), and acoustic impedance (red) and topography (black) along the scan line. Regions of interest (i–vii) related to stages of bone formation (glass granule, Si-layer, HA-layer, epoxy, non-mineralized bone tissue, HA-layer, Si-layer) are indicated in each figure and discussed in detail in corresponding paragraphs. The acoustic impedance (**c**, red line) along the scan line is well explained by the elemental analysis from SEM–EDX (**a**), and the topography maps from SWLI (**b**) and CESAM (**c**) are in good agreement. Thus, CESAM encapsulates the relevant information for bone growth estimation.

To validate the CESAM, a leporine femoral epicondyle with a BAG-S53P4 implant was imaged ex vivo. Quantitative images recorded with SEM (including elemental analysis with EDX), SWLI, and CESAM were manually co-registered (Fig. 1, left). For close inspection, we obtained data about the composition of the sample (SEM–EDX), its surface topography (CESAM and SWLI), and acoustic impedance (CESAM) along one scan-line (Fig. 1). This scan line was chosen to intersect with both the centre of a glass granule and surrounding newly-forming bone, featuring several different materials of interest. Similarities in the structures apparent in the different images allowed the manual co-registration. Regions-of-interest along the scan line (i–vii) were selected as shown in Fig. 1.

### Region (i)

Along the scan line, from 0 to 140 µm, the region shows high silicon content as well as intermediate sodium and calcium content (Fig. 1a). The elemental composition (high Si content, moderate and similar Na and Ca content) matches that of the glass granules in BAG. In the topography maps from both the SWLI (Fig. 1b) and the CESAM (Fig. 1c, black curve), a comparatively flat region is visible. The acoustic impedance (Fig. 1c, red curve) in this region is high, *Z* = 7.7 ± 1.2 MRayl (mean ± 1 SD). The dip in acoustic impedance at the very beginning of Region (i) is caused by a small surface defect also visible in both topography maps. Excluding this dip yields an acoustic impedance for Region (i) of *Z* = 8.0 ± 0.9 MRayl. The high acoustic impedance confirms the presence of a hard material, i.e. glass. This is further supported by the presence of a flat surface in both SWLI and CESAM topography maps.

### Region (ii–iii)

As the BAG is implanted, a thick Si-layer is formed immediately at the glass surface, onto which a thin HA-layer, containing CaP, is formed^36,37^. In Region (ii) (140–160 µm), the SEM–EDX shows a spike in Si and C along with a gradual increase in both Ca and P. This gradual increase in Ca and P, starting in Region (ii) and continuing into Region (iii), supports the hypothesis that the HA-layer is located at the border between Regions (ii) and (iii). Bone formation occurs gradually on top of the HA-layer, which is indicated by constant Ca and P contents accompanied by decreasing Si-content and increasing C-content in Region (iii) (160–205 µm). The acoustic impedance of the flat Region (iii) shows a moderate acoustic impedance, reaching *Z* = 5.4 ± 0.3 MRayl, with a curve shape resembling the Si-, Ca-, and P-content. Both the SWLI and the CESAM topography maps show a notch, containing a minute particulate, in Region (ii) between the glass granule and the surrounding bone formation layer. Similar narrow notches are also visible between all other glass granules and their surrounding bone formation layers. The apparent low acoustic impedance in Region (ii), *Z* = 1.8 ± 0.2 MRayl, which is based on the amplitude of the reflected echo, can be explained by significant sound scattering in the vicinity of this narrow notch.

### Region (iv)

SEM–EDX shows a high C content in Region (iv) (205–320 µm) with no Si, Ca, P, or Na, and CESAM shows a low acoustic impedance, *Z* = 2.5 ± 0.3 MRayl (for comparison, the acoustic impedance of water is 1.5 MRayl). These properties could be indicative of the presence of connective tissue. However, the homogeneity of the acoustic impedance map in this area (Fig. 1c, left) suggests that the material is not tissue, but the fixing agent epoxy. Similar homogeneous patches are seen elsewhere between glass granules in the impedance map, supporting this proposition. The topography maps from both SWLI and CESAM further support this, as they show a smooth ridge ~ 5 µm higher than the surroundings. Connective tissue would not form such a smooth ridge. This ridge could be the product of insufficient polishing time with the initial coarse sandpaper of the sample. The sample was polished with P4000 sandpaper in the final polishing phase, which should remove sharp edges, but a smooth ridge has remained.

### Region (v–vi)

Region (v) (320–375 µm) displays an increase in Ca and P, which indicates the presence of bone tissue. In this region, the shape of the acoustic impedance curve follows the shape of the Ca- and P-curve. The low acoustic impedance, peaking at *Z* = 3.2 MRayl simultaneously with Ca and P, suggests that this tissue has not yet mineralized. The topography maps from SWLI and CESAM differ somewhat in this area; both display a notch in Region (vi) (375–395 µm), but the flatter region in Region (v) is almost 1 µm higher when measured with CESAM. The notch is also visible in SEM–EDX. At the right edge of Region (vi), the Si, Ca and P levels begin to increase, as the line starts intersecting the bone formation layer around granule (B).

### Region (vii)

In the left part of Region (vii), from 395 to 435 µm, the Si content continues to increase, and C, Ca and P are present. This is accompanied by an increase in acoustic impedance, reaching Z = 5.4 MRayl, again in phase with the Ca and P content, similar to that seen in Region (iii). This suggests bone formation on top of the HA-layer of granule (B), containing Si and CaP. At 450 µm, CaP is no longer present and Si has further increased, which suggest that the scan line intersects the thick Si-layer around granule (B) in a tangential direction. In Region (ii), the scan line intersects the Si-layer around granule (A) radially for a short distance and the notch induces scattering, resulting in an apparent low acoustic impedance. In Region (vii), the acoustic impedance estimate from 435 µm to the end of the line is more reliable (both the CESAM topography map and SWLI show a flat region). The acoustic impedance in this Si-layer is *Z* = 3.1 ± 0.4 MRayl.

Thus, most of the different materials expected to be present along the scan line have significantly different acoustic impedances, allowing tissue/material identification: glass granules (*Z ≈* 8 MRayl) (Region (i)), bone formation on top of the HA-layer (*Z* = 5.4 ± 0.3 MRayl) (Region (iii)), and Si-layer surrounding the glass granules *Z* = 3.1 ± 0.4 MRayl. The non-mineralised bone tissue in Region (v) (*Z* = 2.4 ± 0.4 MRayl) and the epoxy in Region (iv) (*Z* = 2.5 ± 0.3 MRayl) have similar acoustic impedances, but the homogeneity of the epoxy makes it distinguishable from non-mineralised bone tissue.

### ROI determination from simultaneous acoustic impedance and topography maps

**Figure 2:**
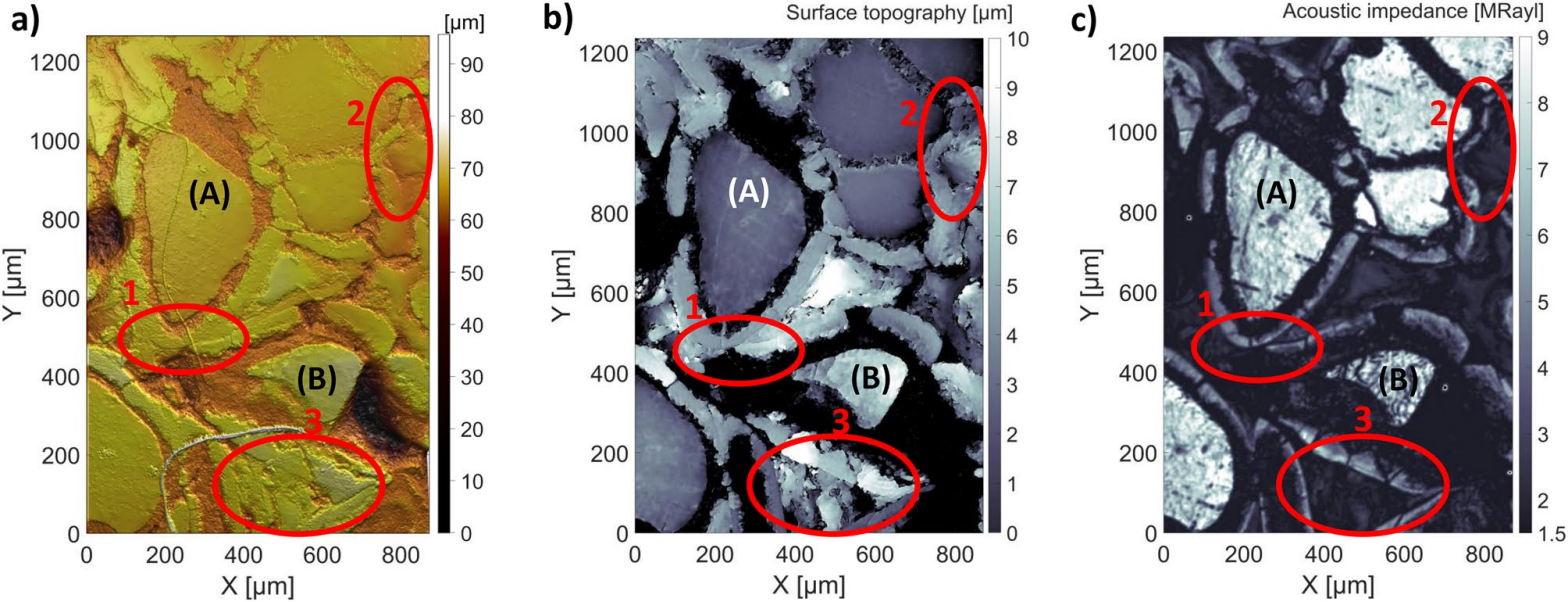
Comparison of (**a**) SWLI image, (**b**) CESAM topography map, and (**c**) CESAM acoustic impedance map to determine regions-of-interest (ROIs) related to bone formation. Three areas (Area 1–3), showing ambiguous features in the topography maps, are indicated in all images. The acoustic impedance information in these regions assists in determining ROIs. Glass granules (A) and (B) are indicated.

As the CESAM provides both an acoustic impedance map and a topography map, it facilitates determining regions-of-interest (ROIs) related to bone formation with higher certainty and ease than with only SWLI or other topography-based techniques. To demonstrate this, three areas (Area 1–3) showing ambiguous features in the topography maps of both the SWLI (Fig. 2a) and CESAM (Fig. 2b) are highlighted in Fig. 2.

### Area 1

Area 1 illustrates the difficulty in distinguishing borders between bone formation layers surrounding glass granules (A) and (B) from topography maps. From the acoustic impedance map (Fig. 2c), it is evident that both granules are surrounded by a bone formation layer (hard boundaries surrounding the glass granules, *cf*. Region (iii), Fig. 1) and the border between the layers is easily distinguishable. In both topography maps (SWLI, Fig. 2a, and CESAM, Fig. 2b), the exact border between the two boundary layers is imperceptible, despite the improved lateral resolution of SWLI compared to CESAM.

### Area 2

In both topography maps (SWLI, Fig. 2a, and CESAM, Fig. 2b), Area 2 shows a flat feature similar to the two adjacent glass granules (to the left). This could indicate another glass granule. However, when examining the acoustic impedance map, one can determine that the two adjacent larger granules are glass (high acoustic impedance, *cf*. Region (i), Fig. 1), while the smaller feature is a different material. Based on the acoustic impedance value and the acoustic homogeneity of the smaller feature, one can conclude that it is epoxy (*cf*. Region (iv), Fig. 1).

### Area 3

Area 3 constitutes an uneven region, whose interpretation from topography maps is elusive. From SWLI (Fig. 2a) or CESAM (Fig. 2b) topography maps, one can only conclude that this region is not a flat glass granule. The acoustic impedance map (Fig. 2c), on the other hand, provides valuable information: The edges of Area 3 comprise bone formation layers around glass granules (hard boundaries surrounding the glass granules, *cf*. Region (iii), Fig. 1), whereas the middle contains softer material (low acoustic impedance). The inhomogeneity of Area 3 further suggests that the material is not epoxy (*cf*. Area 2), leading to an interpretation that it could be connective tissue or non-mineralised bone.

The presented results show that not only does the CESAM acoustic impedance map correspond expectedly with SEM–EDX elemental analysis, but that the CESAM topography map agrees with the SWLI topography map as well. The CESAM topography map is accurate enough to display narrow notches (Region (ii) and (vi), Fig. 1)) that might confound the acoustic impedance values. Hence, CESAM provides an internal reference showing where acoustic impedance values should be treated carefully. Furthermore, utilizing information from both the acoustic impedance and topography maps, generated simultaneously by the CESAM, allows determining regions-of-interest related to bone formation around the BAG with greater confidence and ease (Fig. 2).

## Discussion

As the use of bone substitutes becomes more common, research into the bone formation encompassing them is needed. BAG-S53P4, with its osteoconductive, osteostimulative, angiogenetic^4,5^, and antibacterial properties^6,7^, is of particular interest. However, as the HA-layer forming on the glass granules is indistinguishable from bone mineral in X-rays, the remodelling process is difficult to assess. The proposed SAM method provides a tool for estimating bone formation ex vivo, due to the mechanical contrast between glass granules, HA-layer and forming bone tissue, which is visible in the acoustic impedance map. While SAM can only be used ex vivo for this purpose, it still enables research into the bone formation process on a micron scale in animal models, which can provide important information. Ideally, the information obtained by SAM ex vivo measurements could be used to construct models of bone healing for BAG bone substitutes in the future. These might enable designing in vivo acoustic imaging methods for bone growth evaluation.

SAM, especially coded-excitation SAM (CESAM) as was used here, is capable of fast imaging of quite large areas (a few mm^2^) with a lateral resolution of a few microns^26,34,35^. In most SAM applications, several C-scans (acoustic impedance/topography of a 2D plane) are imaged and then averaged. With CESAM, the imaging speed is significantly higher, as the increased SNR reduces or even eliminates the need for averaging^26^. Another benefit of SAM is that it concurrently provides both an acoustic impedance and a topography map. Having both maps, from a single instrument, of large areas and with high resolution, assists in evaluating regions-of-interest in the samples (Fig. 2). While SAM has been used to image bone previously (e.g.^12,13,18–22,38^), this study is the first to study bone formation with BAG substitutes using a single-C-scan approach and is a continuation of our work described in^34^—the first SAM study of bone formation with BAG substitutes. Furthermore, as the CESAM used in this study improves SNR, it abets imaging of samples with low mechanical contrast^26^.

The advantage of SAM over topography-based techniques is the obtained mechanical contrast from the acoustic impedance map. However, SAM-obtained acoustic impedance values depend on several factors, e.g. tilt, scattering, and frequency. The frequency and focusing of the transducer affect the lateral and axial size of the focus, which affects the reflected echoes. The impact of these effects, their minimisation, and comparison of our impedance values to those presented in the literature are discussed in the following paragraphs.

In this study, a single C-scan of a rectangular area of the sample surface was performed with CESAM. Normal incidence was assumed between the transducer axis and sample surface. This assumption enables straight-forward acoustic impedance estimation from amplitude changes in the reflected echoes caused by different materials present in the sample. However, a tilted surface can deflect part of the reflected acoustic energy past the transducer, causing a drop in amplitude. To ensure that the sample surface was properly aligned to the focal plane of the transducer, a series of B-scans was performed and the sample angle subsequently adjusted with a goniometer. A B-scan shows the Time-of-Flight along one line across the surface. Tilt was removed by ensuring the B-scans in both lateral directions, over long scan lines, showed a straight line. As can be seen from the topography along the line in Fig. 1c, the surface is uneven, but there are flat sections (Region (i), (iii) and (vii)), which on average show no tilt. Also, a tilted sample would show that one edge of the CESAM topography map (Fig. 2b) would be elevated compared to the other. This would be seen in Fig. 2b as a lighter-to-darker colour gradient across the entire sample, which is absent. Figure 2b (SAM topography map) therefore shows that the sample was well aligned. Local height differences and tilts within the sample might also affect the reflected amplitude. However, the depth of focus of our ultrasound transducer was 49 µm (− 6 dB, in water), and therefore the ± 10 µm height variations were well within the focus. Narrow and steep notches, as in Region (ii) and (vi), Fig. 1c, might still cause artefacts in the recorded acoustic impedance. Such notches cause very local and large drops in amplitude (in only a few measurement points), as most of the acoustic pulse is reflected away from the transducer.

In addition to tilt and height variations, surface roughness causes scattering, which affects the reflected amplitude. Especially with high-frequency ultrasound, careful sample preparation and alignment is vital^19,20^. Therefore, for C-scans, the samples should be flat and well-polished. However, many interesting samples, especially biological ones, cannot be polished to a mirror finish. Polishing heterogeneous samples comprising varying mechanical properties presents problems, as softer materials are removed with less effort than stiff ones^19^. Hence, some surface roughness will always remain. To properly account for surface roughness, a multi-layer analysis technique should be used, where the sample is imaged in several planes along the axial direction^19,34^. This allows accounting for local inclinations^19^ or reconstructing the image from several image planes, hence ensuring that each sample point has been imaged at least once in focus^34^. Neglecting scattering, as in our C-scan, tends to decrease the acoustic impedance estimate in regions and samples with high heterogeneity^19^. The benefit of using a single C-scan is that it is fast and requires less post-processing than the aforementioned techniques. In Hyvönen et al.^34^, our CESAM was used to image both fixed and non-fixed leporine femoral BAG samples in several layers, because the height differences across the samples were in excess of 100 µm, i.e. much larger than the depth of focus. For studying the bone formation process around a BAG-implant, using a single C-scan with our CESAM seems sufficient, as the image contrast in both acoustic impedance and topography maps of the single- and multilayer approaches are comparable^34^. A single C-scan with CESAM provides enough contrast to discern areas of different mechanical properties and areas of interest, as shown in Fig. 1 and Fig. 2.

A higher frequency of SAM produces a tighter focus, thereby improving lateral resolution. Raum et al. 2004^20^ demonstrated that a lower frequency (25 MHz) SAM produced lower impedance values of human femoral cortical bone (e.g., 20-year-old female, Z = 7.5 ± 0.2 MRayl), than those recorded with a 50 MHz and a 100 MHz SAM (20-year-old female, *Z* = 8.1 ± 0.3 MRayl and *Z* = 8.4 ± 0.3 MRayl, respectively). The large focal width of the 25 MHz SAM (150 µm) made it impossible to separate the Haversian canals (typical width 45–65 µm^13^) from the bone matrix, causing averaging and apparent lowering of the acoustic impedance, dependent on number, size, distribution, and content of the canals. On the other hand, when high-frequency SAM (900 MHz) is used, there are other effects that cause lower apparent impedance values^18,19^. While the tight focus of a high-frequency SAM improves resolution, scattering caused by surface roughness and local inclinations has an ever-larger impact on the acoustic impedance estimation. In Raum et al. 2003^19^, this effect was studied using high-frequency SAM (900 MHz) of proximal cortical bone from human femora. Three methods were compared: (1) ignoring inclination, i.e. assuming normal incidence, (2) using a mask removing points with > 10° local inclination, and (3) extrapolation from angular-dependence plots. These methods produced average acoustic impedances of osteons Z = 3.9 MRayl, Z = 4.4 MRayl, and Z = 5.10 MRayl, respectively. The authors concluded that neglecting local inclination and surface roughness tends to decrease acoustic impedance estimates, especially with high-frequency SAM. In this study, our CESAM was used with a 256 MHz centre-frequency transducer and coded excitation (linear frequency-modulated chirp, 130–370 MHz, with a Gaussian envelope). This centre frequency presents a compromise between sufficient lateral resolution (5.9 µm) and a depth of focus (49 µm) larger than twice the height variation of the sample. This depth of focus alleviates scattering effects to some degree. The linear chirp concentrates energy into the centre of the band, thus providing high SNR^26^. In conventional SAM, short US bursts are used. Shorter bursts improve axial resolution, while simultaneously reducing the acoustic energy of the back-reflected echo. This causes a trade-off between resolution of the topography map and SNR of the acoustic impedance map. Meriläinen et al.^26^ compared different chirps and a conventional 6-cycle short burst (with comparable bandwidth) with our CESAM on a USAF 1951 resolution sample. Using the 130–370 MHz linear chirp improved both the axial resolution of the topography map (27% decrease in pulse length) and the SNR of the acoustic impedance map (16 dB increase compared to 6-cycle burst)^26^.

As described in the previous paragraphs, acoustic impedance estimation depends on several factors. Thus, a comparison of our impedance values to those found in the literature is necessary. The acoustic impedances related to bone formation in Fig. 1c, as measured with our 256 MHz SAM, were *Z* = 5.4 ± 0.3 MRayl and *Z* = 4.9 ± 0.3 MRayl for the bone mineral on the HA-layer forming around the BAG granules in Regions (iii) and (vi), and *Z* = 2.4 ± 0.4 MRayl for the probable non-mineralized bone tissue in Region (v). The acoustic impedances for the, at least partly, mineralised bone in our leporine femoral epicondyle are similar to those measured by Schulz et al. for newly formed bone tissue in leporine femoral condyles (averages in the range *Z* = 4.2–6.9 MRayl)^39^. Our values are also similar to average acoustic impedances of osteons in human femoral cortical bone measured with 900 MHz SAM^19^. Raum et al. 2003 measured *Z* = 5.10 ± 0.05 MRayl, when compensating for scattering by extrapolation from angular-dependence plots^19^. However, the acoustic impedances of femoral cortical bone in^19^ are low compared to other studies, probably due to underestimation of tilt and hence impedances, when using high-frequency SAM^19^. As mentioned, Raum et al. 2004^20^ demonstrated how 25 MHz SAM produced lower impedance values than the 50 and 100 MHz SAM, but even with 25 MHz SAM, the acoustic impedance of a 76-year-old female (with the lowest acoustic impedance of the study) was *Z* = 7.2 ± 0.2 MRayl. A study of human cortical bone in radii using 200 MHz SAM also produced higher impedance values, in the range 7.2–9.3 MRayl^12^. However, as the bone tissue in our study is from a leporine femoral epicondyle, consisting of trabecular bone, and furthermore is only in the process of bone formation, it is reasonable that we obtain lower impedances. Acoustic impedances of human trabecular bone measured with SAM are in the range *Z* = 6.2 ± 0.6 MRayl (50 MHz, calcanei)^38^ and *Z* = 6.1 ± 0.6 MRayl (100 MHz, femoral neck)^21^, when samples are embedded in poly(methyl methacrylate). Fresh trabecular bone samples have impedances as low as *Z* = 3.5 ± 0.3 MRayl to *Z* = 3.7 ± 0.5 MRayl, when polished with grit P1000 and P4000 sandpaper, respectively^21^. Our sample was not fresh, but had first been stored in formalin at + 4 °C prior to embedding in epoxy, polished with P4000 sandpaper, and lastly dried in a vacuum oven (40 °C) for four weeks. However, if the measured bone tissue is not yet mineralised, the acoustic impedance might even resemble that of cartilage. Cartilage measured in human tibia with 50 MHz SAM had an acoustic impedance of *Z* = 2.12 ± 0.02 MRayl^22^. Hence, our obtained acoustic impedances of non-mineralised and partly mineralised bone (from *Z* = 2.4 ± 0.4 MRayl to *Z* = 5.4 ± 0.3 MRayl and *Z* = 4.9 ± 0.3 MRayl) are in reasonable agreement with the literature.

The main purpose of this proof-of-concept study was to determine the feasibility of the CESAM single-C-scan approach for studying BAG samples, i.e., to validate the acoustic impedance map against SEM–EDX and the topography map against the SWLI map. Despite the difficulty in obtaining reliable absolute acoustic impedance values, the CESAM produced an acoustic impedance map with enough mechanical contrast to distinguish between different tissues, the surrounding epoxy and the glass granules. Identifying ROIs was further aided by the simultaneously measured topography map. Future study directions would be to investigate the bone formation process at different stages of healing. This could also entail a statistical analysis of the presence of different tissue types in the samples and an estimate for uncertainties in acoustic impedance values based on the local flatness of the topography map. Another interesting line of inquiry would be to image fresh samples. For example Ojanen et al.^21^ imaged fresh bone samples, and the ability to image wet leporine BAG samples has already been demonstrated with our CESAM^34^. As our SAM uses coded excitation, it would be interesting to see if the increased SNR and axial resolution could reduce the need for sample polishing, especially if combined with imaging of multiple focal layers.

## Conclusions

We established a method for investigating the bone healing process around BAG implants ex vivo. While X-rays cannot distinguish BAG from the forming bone, coded-excitation SAM produces sufficient mechanical contrast to differentiate between different bone tissue stages, BAG granules, and the surrounding epoxy. The acoustic impedance map of SAM was compared against SEM–EDX and the topography map against SWLI. SAM produces both acoustic impedance and topography maps simultaneously, which simplifies determining regions-of-interest relating to bone formation. CESAM, therefore, constitutes a promising stand-alone tool for investigating bone remodelling around BAG ex vivo.

## Materials and methods

### Bioactive glass

The BAG implants were manufactured as described in^40,41^. BAG-S53P4 (in wt.%: 53% SiO_2_, 23% NaO, 20% CaO and 4% P_2_O_5_) was melted from analytical reagents (Na_2_CO_3_, CaCO_3_ and CaHPO_4_ × 2H_2_O, Belgian quartz sand) at 1360 °C for 3 h in a platinum crucible. To achieve homogeneous BAG, the glass was melted twice. The melt was cast as a block in a graphite mould, then annealed for 4 h at 520 °C and finally cooled overnight in the annealing furnace. The BAG block was crushed and sieved into 300–500 µm granules. The granules were sintered in a graphite mould in nitrogen atmosphere at 720 °C for 90 min to cylinder-shaped porous scaffolds of 5 mm × 15 mm size (diameter x height).

### Bone sample

A direct lateral approach to the knee and exposure of the femoral lateral epicondyle was performed under aseptic conditions on a skeletally mature rabbit (NZW, Harlan laboratories) under general anaesthesia (medetomidine hydrochloride s.c. + ketamine hydrochloride s.c.). A horizontal bone defect of 6 mm was drilled without penetrating the medial cortex. The defect was filled with the 5 mm × 15 mm (diameter x height) scaffold of BAG-S53P4. Cefuroxime, buprenorphine, and carprofen were given postoperatively for 3 days for infection prophylaxis and pain relief. The animal was euthanized at 8 weeks post treatment with an overdose of pentobarbital. The distal part of the femur was cut and stripped from soft tissues and stored in formalin at + 4 °C. The Animal Experimental Board of Finland approved the study (ESAVI/440/04.10.07/2014) and the laboratory animal care guidelines of the University of Helsinki, the ARRIVE guidelines, and the Directive 2010/63/EU of the European Parliament and the Council of the European Union were strictly followed in all aspects of the project.

The bone sample was prepared as described in^41^. The sample was first moulded in epoxy and then ground in ethanol in the axial plane to the centre of the scaffold with increasingly fine sandpaper, lastly with P4000 sandpaper. The sample was subsequently dried in a vacuum oven (40 °C) for four weeks.

### Scanning acoustic microscope

**Figure 3:**
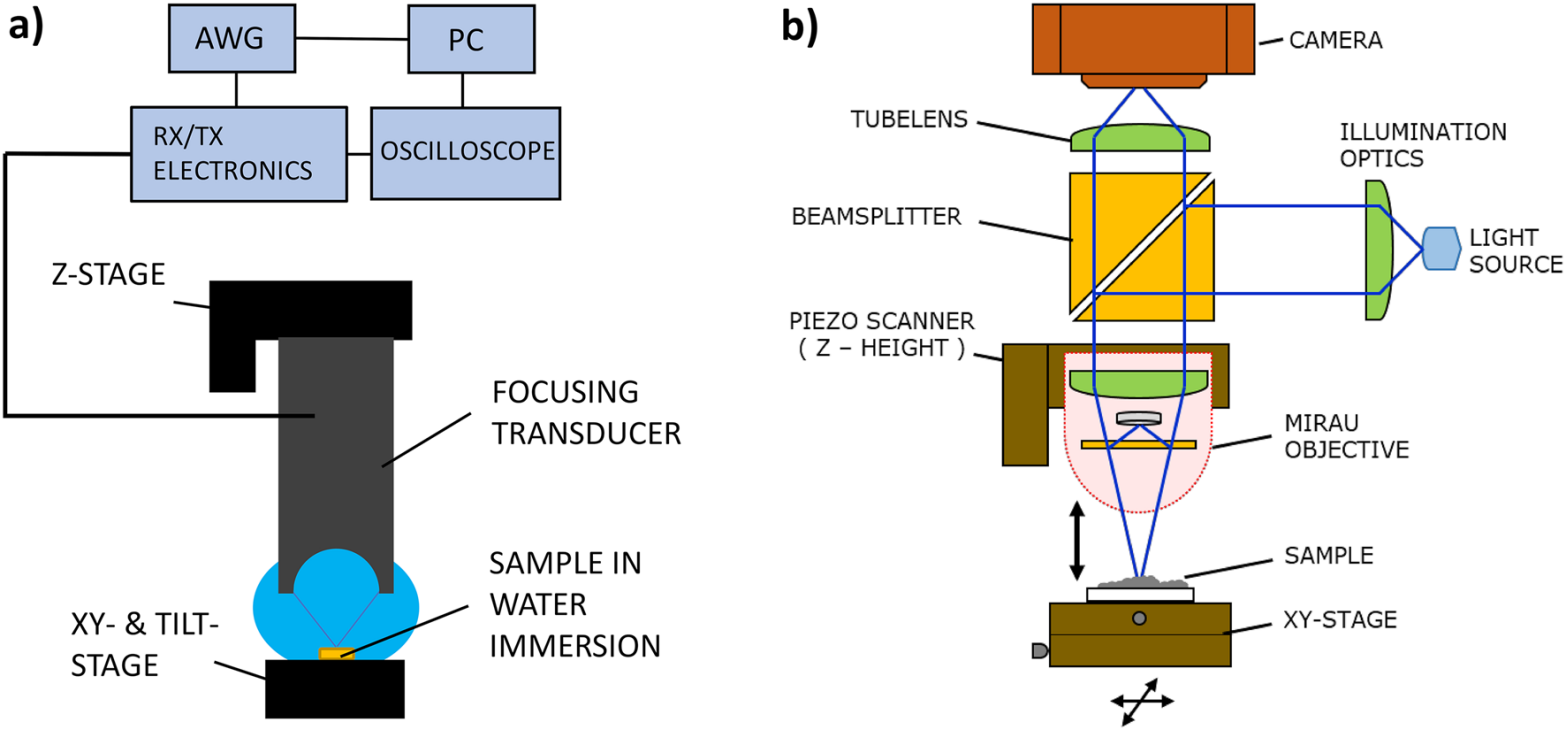
Schematics of (**a**) CESAM and (**b**) SWLI. (**a**) The focused transducer transmits a frequency-modulated coded signal (linear chirp, 130–370 MHz), which is reflected from the sample surface, recorded with the same transducer and post-processed. Both topography and acoustic impedance maps are obtained from one scan. The sample is scanned in the XY-plane in water immersion. (**b**) The light is divided within the Mirau-type objective to two interfering optical paths: scanning and fixed reference. The resulting interference images are recorded by a camera as a function of piezo-controlled objective-to-sample distance, which are used to calculate the topographic map of the sample.

A custom-built CESAM^26^ was used to characterize the sample (Fig. 3a) (transducer centre frequency 256 MHz, lateral width of focus 5.9 µm, depth of focus 49 µm, working distance 577 µm, − 6 dB bandwidth: 144–368 MHz). The transducer was translated horizontally using two orthogonally aligned translation stages (MLS203-1, controller BBD202, Thorlabs, New Jersey, USA) with 50 nm encoder resolution. The transducer was excited with a linear FM chirp signal, 130–370 MHz, with a Gaussian envelope. The chirp was selected based on^26^ for improved SNR and axial resolution. The ultrasonic echo was received with the same transducer, amplified by a low-noise pre-amplifier (ZFL-1000LN+, Mini Circuits, New York, USA) and recorded with a PCIe digital oscilloscope (M4i.2233-x8, 2.5 GS/s Spectrum Instrumentation GmbH, Grosshansdorf, Germany). The scanned area was 0.88 mm × 1.26 mm, and 1 µm stepping was used. Imaging was performed in water immersion.

The acoustic reflections from the top surface of the sample provided the Time-of-Flights producing the topography map. Simultaneously, the amplitude changes of the reflected echoes provided the elastic contrast of the sample comprising both bone and BAG. At each scanned point, the amplitude of the reflected echo depends on the acoustic impedance mismatch at the water-sample interface. To obtain the acoustic impedance of the leporine bone sample, the acoustic amplitude was calibrated using a calibration method described in^19^. All samples were carefully aligned to allow assuming normal incidence of the acoustic pulse at each scanned point. At normal incidence, the reflection coefficient of the acoustic pressure, i.e., the ratio of the reflected pressure amplitude to the transmitted pressure amplitude, is:

1 $$R = \frac{{Z_{sample} - Z_{water} }}{{Z_{sample} + Z_{water} }}$$

where *Z*_*water*_ and *Z*_*sample*_ are the acoustic impedances of water (1.5 MRayl) and the sample (in each measured point). Three different calibration samples were used with the same transmission settings as with the leporine bone sample: acrylic (PMMA), silicon, and sapphire, with acoustic impedances *Z* = 3.3 MRayl, 22.0 MRayl and 41.7 MRayl, respectively. These calibration materials were selected because they have well-defined acoustic impedances and are available as flat samples. The surfaces of the aligned calibration samples were measured with different defocus distances and the focal amplitude (maximum amplitude) was used in the calibration calculations. The amplitudes of the reflected echoes from the bone sample were subsequently compared to the calibrated amplitudes to obtain the acoustic impedance map in Fig. 1c and Fig. 2c. Normal incidence was assumed within each measurement point also for the carefully aligned bone sample. As the focal width was 5.9 µm, this is a fair assumption, except in the vicinity of the narrow notches (Regions (ii) and (iv)).

### SWLI

A custom-built SWLI (Fig. 3b)^31^ was used to image the sample comprising both bone and BAG. The system is built on a Nikon side-illuminated microscope frame. A CMOS camera (Hamamatsu Orca-Flash 2.8, Hamamatsu, Japan) records images through a 10 × objective (10X Nikon CF IC Epi Plan DI, Tokyo, Japan) and through a 1 × tubelens (Nikon 200 mm, Tokyo, Japan). The vertical scanning of the sample was done by moving the objective inside a 100 µm range with a piezo actuator (PI Pifoc P-721-CDQ, Physik Instrumente (PI) GmbH & Co. KG, Karlsruhe, Germany). The sample was scanned in the lateral direction with a xy-translation stage (8MTF-102LS05, Standa Ltd, Vilnius, Lithuania) having a range of 10.2 cm and resolution of 0.31 µm. Spatial overlapping (30%) of sub-aperture scans was used. Data acquisition and surface detection was performed with custom-made software and surface stitching and 3D analysis was done with the commercial software MountainsMap (Digital Surf).

The sample was SWLI imaged with 17 sub-aperture scans and stitched together. As the SWLI is based on reflected light from the sample surface, it has measurement limitations with steep topographical slopes, which tend to scatter most of the light. This can be seen as increased measurement noise on the slopes of steep crevices/protrusions resulting in noisy surface detection in the steep edge areas.

### Scanning electron microscopy (SEM) and EDX elemental analysis

SEM images were obtained with a scanning electron microscope (Coxem SEM EM-30 AX Plus) using an in-lens upper secondary electron (SE) detector (magnification 150x). The sample was coated with a 5 nm Pt/Pd layer. The acceleration voltage was 15.0 kV with a take-off angle of 25.8° and resolution 133 eV (at 5.89 keV Mn). The elemental analysis was performed with an Edax EDX detector of SDD type. The elemental analysis was applied along a 450 µm long scan-line (Fig. 1).

### Co-registration of images

The same area of the sample was imaged with SWLI, SEM combined with EDX, and CESAM. The images were manually co-registered and the image contrast from SWLI (topography) and CESAM (acoustic impedance and topography) were obtained along the same scan line as the elemental analysis (Fig. 1). The SEM, SWLI, and SAM images are all based on different contrast methods, which is challenging for automated algorithm-based co-registration methods. Therefore, the co-registration was performed manually.

## Data Availability

The data presented in this study are available on reasonable request from the corresponding author.
